# In Situ Surface Modification of Paper-Based Relics with Atmospheric Pressure Plasma Treatment for Preservation Purposes

**DOI:** 10.3390/polym11050786

**Published:** 2019-05-02

**Authors:** Xu Yan, Guo-Sai Liu, Jing Yang, Yi Pu, Shuo Chen, Hong-Wei He, Conger Wang, Yun-Ze Long, Shouxiang Jiang

**Affiliations:** 1 Industrial Research Institute of Nonwovens and Technical Textiles, College of Textiles and Clothing, Qingdao University, Qingdao 266071, China; rl19900628@163.com (G.-S.L.); 15764238363@163.com (J.Y.); puyiconan@163.com (Y.P.); shuochensd@163.com (S.C.); hhwpost@163.com (H.-W.H.); yunze.long@163.com (Y.-Z.L.); 2 Collaborative Innovation Center for Eco-Textiles of Shandong Province, Qingdao University, Qingdao 266071, China; 3 Institute of Textiles and Clothing, The Hong Kong Polytechnic University, Hong Kong, China; 4 State Key Laboratory of Bio-Fibers and Eco-Textiles, Qingdao University, Qingdao 266071, China; wangcedaily@163.com; 5 Collaborative Innovation Center for Nanomaterials & Optoelectronic Devices, College of Physics, Qingdao University, Qingdao 266071, China

**Keywords:** plasma treatment, paper-based relics, waterproofing, preservation

## Abstract

Paper-based relics, which are an important part of cultural heritage worldwide, are at risk of imminent damage from various environmental sources. To protect them, the atmospheric pressure plasma polymerization of hexamethyldisiloxane (HMDSO) precursor has been explored on paper-based relics in situ. The macro and micro images taken during this process suggest that the in situ plasma treatment does not change the macro morphology and the micro structure of the treated paper-based relic samples. On the other hand, plasma treatment causes the polymerization of the HMDSO which then produces nanoparticles deposited onto the paper-based relics. These nanoparticles provide good waterproof properties with large static water contact angles and smaller rolling angles, which protect the paper-based relics from water penetration. Moreover, since the nanoparticles are deposited onto the fibers, waterproof fastness is ensured. Also, the examined mechanical properties of the treated and untreated paper-based relics indicate that the atmospheric pressure plasma treatment does not affect the strength of the paper very much. The results in this study show that atmospheric pressure plasma treatment with the use of HMDSO precursor is a good method to preserve paper-based relics.

## 1. Introduction

Paper has been the primary type of material for recording information worldwide since its invention. Paper-based relics are an important component of cultural heritage, including rare historical books, calligraphy and paintings, and other culturally- and historically-invaluable objects [1,2,3,4]. However, a large number of paper-based relics are at risk of imminent damage from various environmental sources, with many becoming increasingly fragile and prone to damage with time [1,2,3,4,5]. Therefore, it is important to protect and preserve paper-based documents in libraries, archives, and museums.

The main components of paper-based relics are usually paper, ink, paint, and pigment for written and painted works [4]. However, both internal and external damage factors could negatively impact them [4,5,6,7,8,9,10,11,12,13,14,15]. Since the main components of paper are cellulose, lignin, and hemicelluloses, they can readily hydrolyze and oxidize and, along with the acidic or oxidized inks and pigments, lead to acidification and then a reduction in hardness and folding strength [5,6,7,8,9,10]. This is an internal damage process. Moreover, external damage factors such as temperature, gas, dust, microbial contamination, oxidation, water immersion, visible and ultraviolet (UV) radiation, and mechanical wear and tear may also affect paper-based relics [1,2,3,4,5,12,13,14,15]. For example, UV–Visible light exposure may accelerate the rate of chemical reaction in paper, as well as in the ink and pigments, due to its high energy, and then cause serious ageing and fading [15]. Water on the surface of and inside the paper could increase the rate of hydrolysis of cellulose by providing H^+^ as the catalyst, which also blurs the pigments on the paper [9,14].

Attempts have been made in the field by using both physical and chemical means to protect and preserve paper-based relics. Pasting and framing, silk net lamination, and the parylene process are usually adopted to improve the strength of paper [4,12,14]. To deacidify paper, various mildly-alkaline agents have been used not only to neutralize the acids, but also to deposit an alkaline substance that will neutralize possible future acidity [2,9,10]. To prevent water damage, electrospinning films have been proposed due to their uneven surfaces [14,16,17,18,19,20]. However, these methods still have some unavoidable drawbacks, such as complex procedures, requirements for strict conditions such as a vacuum environment, and a loss of transparency.

Recently, plasma treatment has been promoted as a new approach for modifying the surfaces of textiles and paper. This treatment only changes the surfaces of the materials without affecting their bulk properties [21,22,23,24,25,26,27,28]. In the preservation of paper-based relics, plasma treatment has been proposed for deacidification purposes, with the use of calcium hydroxide (Ca(OH)_2_) as the precursor [26]. It is indicated that plasma treatment does not require the immersion of paper into a solution, does not wrinkle or deform the paper, does not cause changes in color, and has a certain strengthening effect which increases the strength of paper, along with a long-lasting deacidification effect [26]. Moreover, N_2_ and O_2_ plasma treatment has also been used for waterproofing textiles through the atmospheric pressure plasma polymerization of hexamethyldisiloxane (HMDSO) precursor [24]. Furthermore, anti-UV properties have also been attained by applying air low-temperature plasma treatment to textiles [27,28].

We used atmospheric pressure plasma treatment in this study based on its good outcomes on waterproof paper-based relics. Both the static and dynamic water contact angles (WCAs) of the treated paper-based relics were measured, and the effects of the plasma treatment process parameters on these angles were examined. Moreover, scanning electron microscopy was used to analyze the fractured surface of the untreated- and plasma-treated paper-based relics. Finally, the strength of the treated relics was also examined.

## 2. Materials and Methods

### 2.1. Materials

The HMDSO precursor (99%) was purchased from Aladdin (Shanghai, China) and used as received. The so-called paper-based relic used for this study was a piece of Chinese calligraphy on untreated rice paper, which was written by Jinyuan He. Untreated rice paper, which easily absorbs water or ink, was purchased from Taobao market (Anhui, China).

### 2.2. Plasma Polymerization

**Figure 1:**
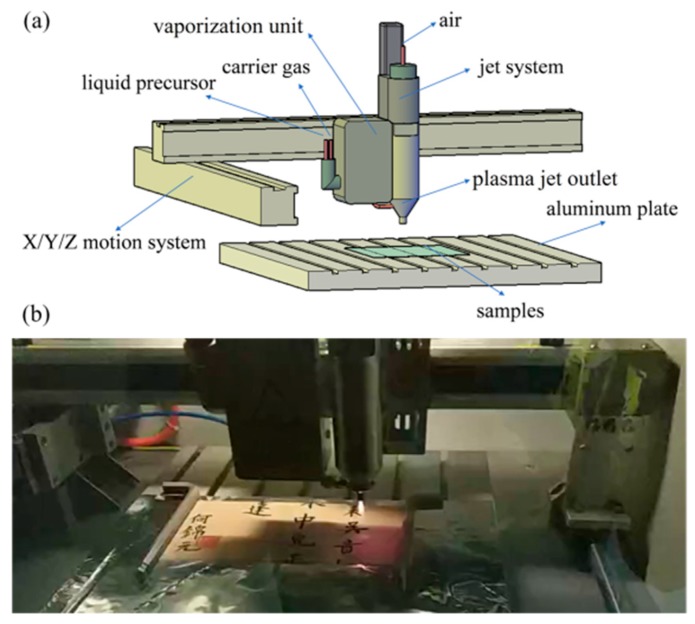
Schematic view of the atmospheric pressure plasma system (**a**) and the real image of the process of atmospheric pressure plasma treatment (**b**).

Plasma polymerization was carried out using the PlasmaTreater AS400 atmospheric pressure plasma jet system with a PFW10 nozzle (PlasmaTreat GmbH; Steinhagen, Germany), as shown in Figure 1. The details of the plasma system are reported in [24]. In this research study, compressed air from the gas bottle was used to produce the plasma with a flow rate of 1500 L/h The HMDSO precursor was passed through the vaporization component and mixed with 300 L/h of the metered carrier gas argon, and the gas flow was discharged through the plasma jet outlet. The paper-based relic samples were fixed onto the aluminum plate in the atmospheric-pressure plasma jet system (Figure 1b), and the plasma jet was controlled using the X/Y/Z motion system. The speed of the jet, the distance between the substrate and the jet nozzle, and the transverse distance were set at 5 m/min, 4 cm, and 2 mm, respectively, for each experiment. Under these conditions, it may take 100 min to cover a 1 m² paper relic. Moreover, in this plasma treatment process, the temperature of the plasma jet was set at about 110 °C, which may have accelerated the ageing of the paper. However, with the above fast jet movement speed and the transverse distance for a regular paper relic (calculated in cm unit), the paper relic was only subjected to 110 °C for a short amount of time. Consequently, the ageing effect can be ignored. The other operational parameters (precursor value, voltage, and number of plasma treatments) for this study are shown in Table 1. All of the experiments were carried out in ambient conditions.

### 2.3. Characterization

Images of the paper-based relic samples were taken with a digital camera (Nikon D7000, Tokyo Metropolis, Japan). The surface morphology of the untreated and treated paper-based relic samples was examined using a scanning electron microscope (SEM) (Phenom Pro, Thermo Fisher Scientific, Phenom-World B.V. 81 Wyman Street, Waltham, MA, 02454, US) operated at an acceleration voltage of 10.0 kV, after being subjected to Au sputtering for 60 s and once the Au film thickness reached about 20 nm. The WCAs of the paper-based relic samples were examined using an optical tensiometer (Attension Theta, Biolin Scientific, Germany) with a water drop volume of 2 μL for three regions at room temperature. An analysis was carried out by drop fitting a Young–Laplace equation through Theta software. Each WCA value was the mean value of five random sites in each of the three regions. Fourier transform infrared spectroscopy (FTIR) spectrums were measured using a Nicolet iN10 spectrometer (Thermo Scientific). The mechanical properties of the untreated and treated paper-based relic samples were tested on a computer-controlled tensile testing system (INSTRON 3699, Shanghai Instron Co., Ltd., Shanghai, China). The color change of the treated sample was examined by X-Rite Color Premier 8400 using the CIE L*, a*, b* system and determined by the equation: $∆E=\sqrt{{\left(∆L^{*}\right)}^{2}+{\left(∆a^{*}\right)}^{2}+{\left(∆b^{*}\right)}^{2}}$.

## 3. Results and Discussion

**Figure 2:**
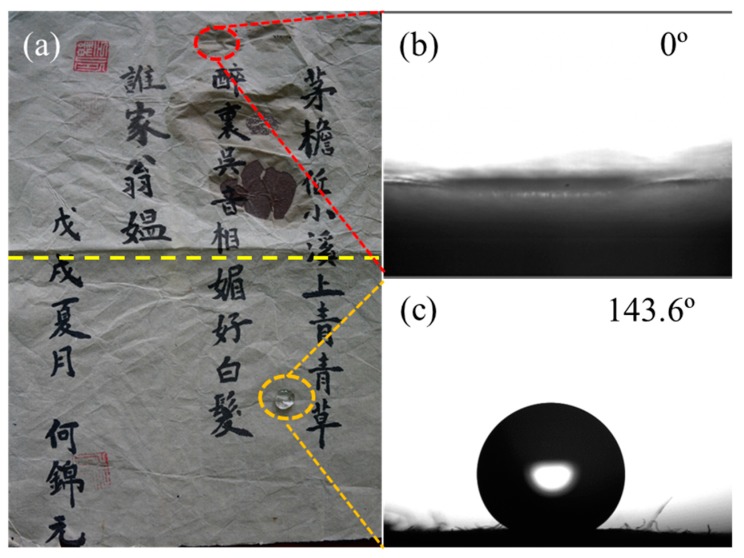
Water drops on the surfaces of the untreated (upper region of the dashed bright yellow line) and treated paper relics (lower region the dashed bright yellow line) (**a**) and the measured water contact angle (WCA) of the untreated (**b**) and treated (**c**) paper relics.

After applying air plasma treatment (300 V) and polymerization of the HMDSO precursor at 30 g/h to the paper-based relic sample (Figure 1b), we then compared the wettability of the untreated and treated paper-based relic samples. As shown in Figure 2a, the water droplet on the untreated region seeped through the paper quickly, and the WCA was zero (Figure 2b). After the plasma treatment, the water droplet was not absorbed into the paper, and the WCA was substantially increased to 143.6°, as shown in Figure 2c. More details can be found in Videos S1 and S2 in the Supplementary Materials. It can also be observed in Figure 2 that the plasma treatment on the paper-based relic sample did not obscure the handwriting. Moreover, the color changes of the treated samples are summarized in Table 2, and one can find that the plasma treatment involved little color change.

**Figure 3:**
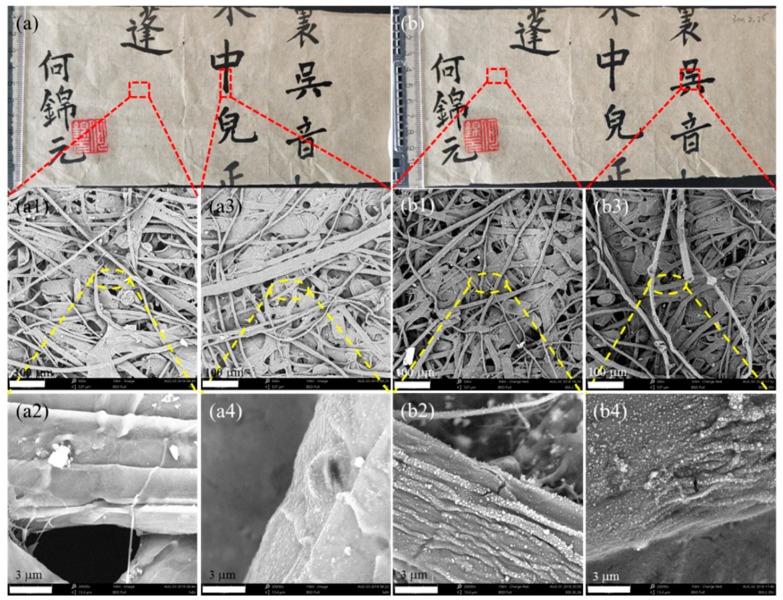
The macro and micro SEM images of the paper relics before (**a**–**a4**) and after (**b**–**b4**) plasma treatment (300 V, 30 g/h, two repeat treatments) at blank and inked sites, respectively.

Figure 3 also suggests that the paper-based relic sample did not show macroscopic changes before and after the plasma treatment. Moreover, the micro SEM images of the paper-based relic sample before and after plasma treatment [Figure 3a1,a3,b1,b3] also suggest that there were no obvious changes in the structure of the fibers, regardless of whether there was ink. As for the single fibers [see Figure 3a2,a4,b2,b4], the treated fibers showed an obviously-increased roughness with some nanoparticle deposition. These nanoparticles were produced from the polymerization of HMDSO during the plasma treatment [24,27,28]. Moreover, the deposited nanoparticles and increased roughness of the fibers probably resulted in the larger WCA and the increased waterproof level [24].

**Figure 4:**
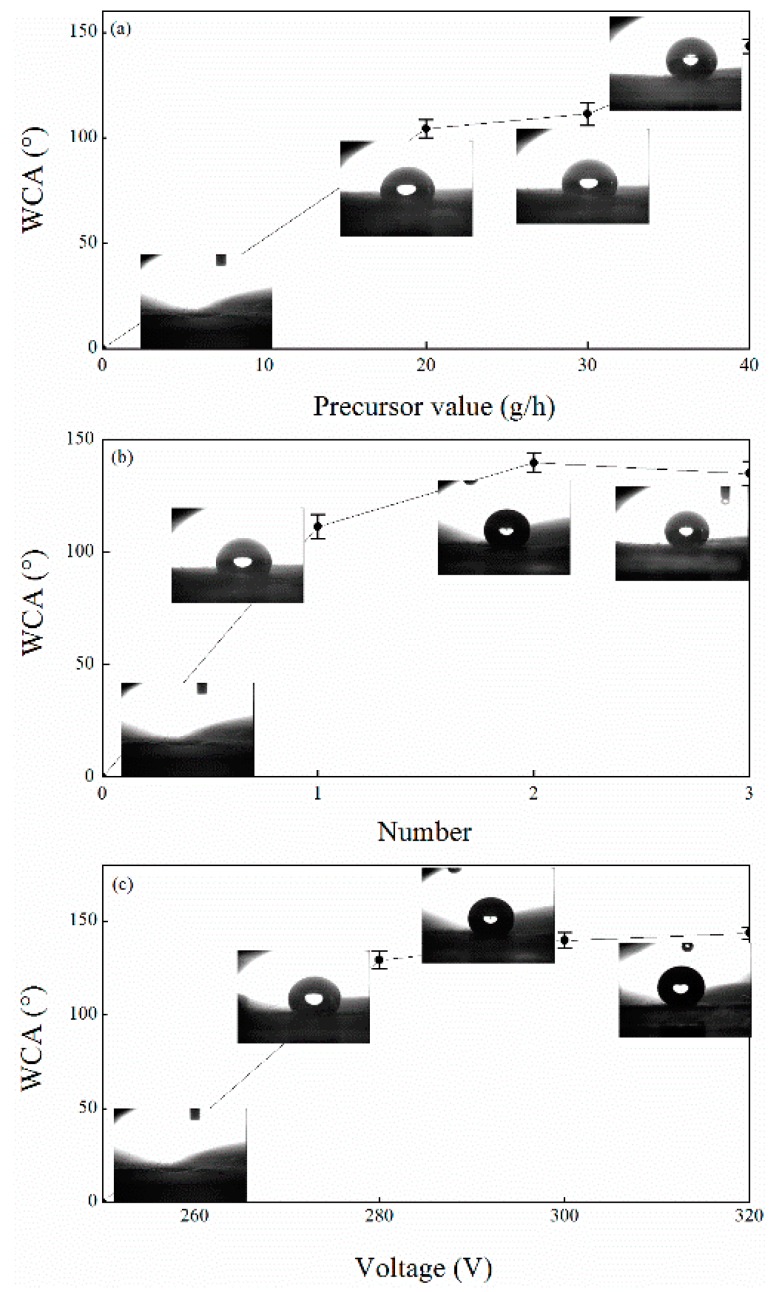
Relationship between the parameters in the plasma treatment process and the WCA under the following conditions: (**a**) voltage 300 V, one treatment, (**b**)voltage 300 V, precursor value 30 g/h, and (**c**) precursor value 30 g/h, two treatments.

The effects of the plasma treatment process parameters on the waterproof properties of the paper-based relic samples were also investigated. As shown in Figure 4a, a higher precursor flow increased the WCA of the sample. This indicates that the precursor flow value may affect the formation and deposition of particles [24,29]. As the precursor flow value increased, more and more HMDSO nanoparticles agglomerated and then formed a larger and denser coating on the surface of the sample, which is shown in Figure S1a1–a3 in the Supplementary Materials. Consequently, a larger WCA was produced, as indicated in Figure 4a. The increased treatment quantity was also found to affect the WCA of the paper-based relic sample. As can be seen in Figure 4b, the second treatment on the paper-based relic sample might have increased the WCA due to the deposition of more HMDSO nanoparticles, while the third treatment reduced the WCA, which could be attributed to the reduction of the HMDSO nanoparticles, as shown in Figure S1b1–b3 in the Supplementary Materials. It can be theorized that after the first two treatments, the HMDSO nanoparticles may cover the surface fibers; and once the third treatment is applied, the high energy of the plasma jets may destroy the covered nanoparticles. Moreover, a higher plasma voltage also provided an obviously-larger WCA on the paper-based relic samples (Figure 4c), which might be the result of the higher energy of the ions, radicals, and electrons in the plasma which then affect the formation and deposition of HMDSO (see Figure S1c1–c3 in the Supplementary Materials). Generally, a larger WCA is closely associated with a higher quantity of HMDSO nanoparticle deposition.

**Figure 5:**
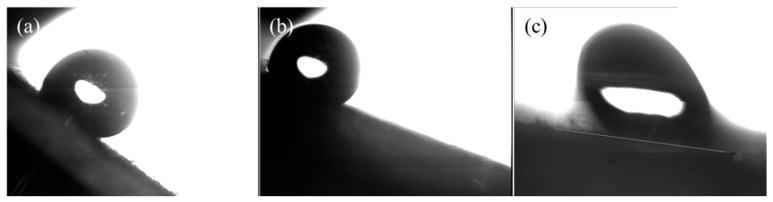
The critical roll angle measured at different precursor values, (**a**) 20 g/h, (**b**) 30 g/h, and (**c**) 40 g/h, with the other conditions being 300 V and two treatments.

Moreover, the movement of the water drop on the treated surface was investigated. As suggested in Figure 5, the roll angle was decreased as the precursor value increased, which may be attributed to the higher WCA. More details could be found from Video S3 in the Supplementary Materials. The smaller roll angle may prevent water staying on the paper relic and thus prevent water invasion.

**Figure 6:**
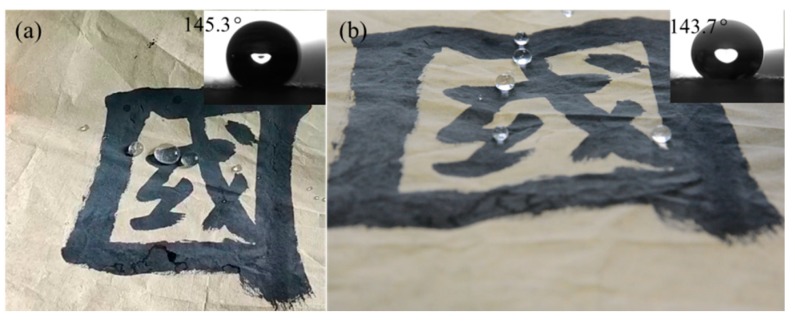
The WCAs of the treated samples under 300 V and 30 g/h, during two measurements: (**a**) soon after treatment, and (**b**) six months later.

Furthermore, the waterproof fastness of the plasma-treated paper-based relics was examined. As can be seen in Figure 6, the WCA of the treated samples was 145.3° soon after the plasma treatment, and the angle decreased to 143.7° after six months. The little change of the WCA on the plasma-treated paper-based relic suggests that plasma polymerization of the HMDSO has waterproof fastness, which may result from the deposition of the HMDSO onto the fibers.

**Figure 7:**
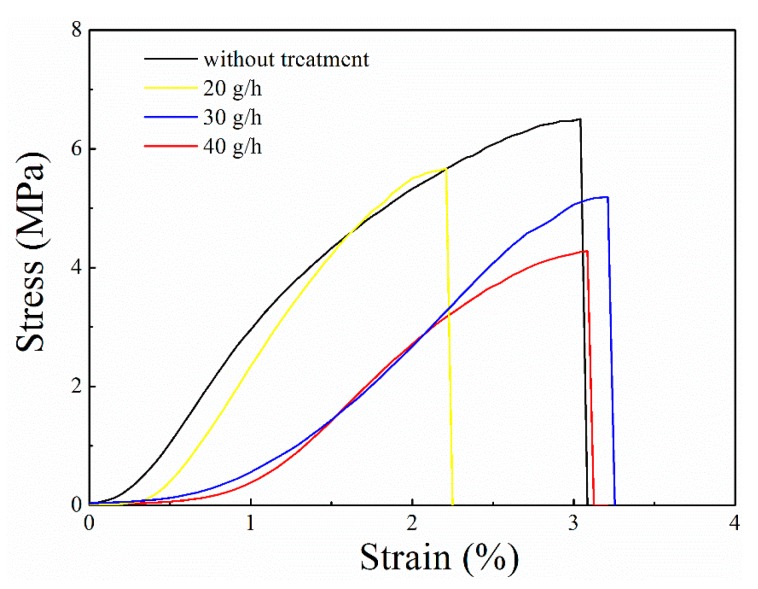
Stress–strain curves of the untreated and plasma-treated paper relics under different parameters (Color online).

It has been pointed out that plasma treatment of the surface of a material (including polymerization) would not damage the mechanical properties of the target because there is little or no penetration [24,30]. The mechanical properties of the untreated and plasma-treated paper-based relic samples with different amounts of precursor were also investigated and are shown in Figure 7. The results show that the stress does not seem to have decreased after the plasma treatment, while the strain might be slightly higher as a result of the treatment. Since paper-based relics, especially Chinese calligraphy and paintings, are often framed [4], the mechanical properties are not a key concern in their preservation. Thus, the slight changes in the mechanical properties after plasma treatment are not consequential.

**Figure 8:**
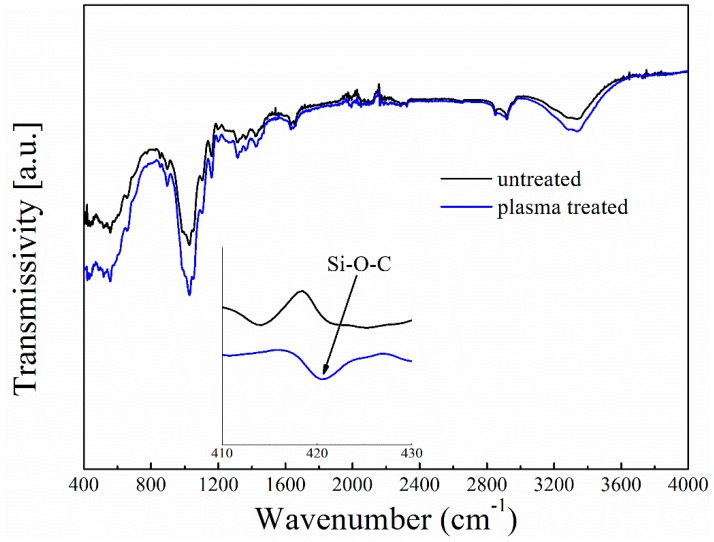
FTIR spectrum of the untreated and plasma-treated paper relics. The inset is the enlargement of the wavenumber region 410–430 cm^−1^.

The FTIR spectra of the untreated and plasma-treated paper-based relic samples (300 V, 30 g/h, two times) were also examined, and are shown in Figure 8. Generally, it could be observed that the plasma treatment did not change the major chemical structure of the paper-based relics, which could be attributed to the small amount of HMDSO deposition and the rapidness of the deposition. However, there were still some local changes due to the deposition of the HMDSO, as shown in Figure 3b and Figure 7, which were a result of the polymerization of the HMDSO during the plasma treatment, and the result was the formation of a new chemical group Si–O–C [24,27,28]. Furthermore, it has also been suggested that the polymerization of HMDSO could form a thin silicon dioxide (SiO_2_ film), which could enhance the anti-UV properties of paper-based relics [27,28].

## 4. Conclusions

In summary, we addressed the treatment of paper-based relics by using atmospheric pressure plasma with an HMDSO precursor. The macro morphology of the plasma-treated paper-based relic samples remained unchanged, as only the surfaces of the samples were modified. On the other hand, polymerized HMDSO nanoparticles were deposited onto the fibers during the treatment process. Consequently, the deposited polymerized HMDSO nanoparticles provided a larger WCA for a longer period of time on the paper-based relic samples, and thus prevented them from water penetration. Moreover, the plasma treatment did not change the mechanical properties and the chemical structure of the paper-based relic samples. These results indicate that atmospheric pressure plasma treatment might be a good method of paper preservation.

## Figures and Tables

**Table 1:** Operational parameters considered in plasma treatment process.

Operational Parameters	Unit	Values
Precursor feeding value	g/h	20, 30, 40
Plasma voltage	V	280, 300, 320
Treatment quantity	-	1, 2, 3

**Table 2:** Color changes of untreated and treated paper-based relics.

CIE Parameters	Blank Area		Inked Area	
	Untreated Sample	Treated Sample	Untreated Sample	Treated Sample
L*	75.96	76.19	26.17	25.25
a*	3.13	3.18	0.42	0.46
b*	20.51	20.97	2.03	2.16
∆E	0.517		0.93	

## Supplementary Materials

The following are available online at https://www.mdpi.com/2073-4360/11/5/786/s1, Video S1: Water drop onto the untreated and treated Chinese calligraphy. Video S2: Water drop onto the untreated and treated Chinese ink-wash painting relic. Video S3: Rolling water drop onto the plasma-treated paper relics. Figure S1: The SEM images of the plasma-treated paper relic with different precursor values: (a1) 20 g/h, (a2) 30 g/h, and (a3) 40 g/h under 300 V, and one-repeat treatment times. Different repeat treatment numbers 1–3 are shown under 300 V and a precursor value of 30 g/h in (b1)–(b3), respectively. Different voltages are also shown with a precursor value of 30 g/h and two repeat treatment times: (c1) 280 V, (c2) 300 V, (c3) 320 V.

## References

[1] Alexopoulou I., Zervos S. (2016). Paper conservation methods: An international survey. J. Cult. Herit..

[2] Zervos S., Alexopoulou I. (2015). Paper conservation methods: A literature review. Cellulose.

[3] Chen Q., Wen W.Y., Qiu F.X., Xu J.C., Yu H.Q., Chen M.L., Yang D.Y. (2016). Preparation and application of modified carboxymethyl cellulose Si/polyacrylate protective coating material for paper relics. Chem. Pap..

[4] Liu J., Wang J. (2010). Main factors affecting the preservation of Chinese paper documents: A review and recommendations. IFLA J..

[5] Daniels V.D. (1996). The chemistry of paper conservation. Chem. Soc. Rev..

[6] Carter H.A. (1996). The chemistry of paper preservation: Part 1. the aging of paper and conservation techniques. J. Chem. Educ..

[7] Carter H.A. (1996). The chemistry of paper preservation: Part 2. The yellowing of paper and conservation bleaching. J. Chem. Educ..

[8] Area M.C., Cheradame H. (2011). Paper aging and degradation: Recent findings and research methods. BioResources.

[9] Baty J.W., Maitland C.L., Minter W., Hubbe M., Jordan-Mowery S.K. (2010). Deacidification for the conservationand preservation of paper-based works: A review. BioResources.

[10] He B., Lin Q., Chang M., Liu C., Fan H., Ren J. (2019). A new and highly efficient conservation treatment for deacidification and strengthening of aging paper by in-situ quaternization. Carbohydr. Polym..

[11] Feng W. (2011). Discussion on reason of ancient books’ moth-eaten and control measures. J. Libr. Inf. Sci. Agric..

[12] Princi E., Vicini S., Pedemonte E., Arrighi V., McEwen I. (2005). New polymeric materialsfor paper and textile conservation. I. Synthesis and characterization of acryliccopolymers. J. Appl. Polym. Sci..

[13] Princi E., Vicini S., Pedemonte E., Arrighi V., McEwen I. (2007). New polymeric mate-rials for paper and textiles conservation. II. Grafting polymerization of ethylacrylate/methyl methacrylate copolymers onto linen and cotton. J. Appl. Polym. Sci..

[14] Li Q., Xi S., Zhang X. (2014). Conservation of paper relics by electrospun PVDF fiber membranes. J. Cult. Herit..

[15] Robotti E., Bobba M., Panepinto A., Marengo E. (2007). Monitoring of the surface of paper samples exposed to UV light by ATR-FT-IR spectroscopy and use of multivariatecontrol charts. Anal. Bioanal. Chem..

[16] Liu M.N., Yan X., You M.H., Fu J., Nie G.D., Yu M., Ning X., Wan Y., Long Y.Z. (2018). Reversible photochromic nanofibrous membranes with excellent water/windproof and breathable performance. J. Appl. Polym. Sci..

[17] Liu G.S., Yan X., Yan F.F., Chen F.X., Hao L.Y., Chen S.J., Lou T., Ning X., Long Y.Z. (2018). In Situ electrospinning iodine-based fibrous meshes for antibacterial wound dressing. Nanoscale Res. Lett..

[18] Cai M., He H., Zhang X., Yan X., Li J., Chen F., Yuan D., Ning X. (2019). Efficient synthesis of PVDF/PI side-by-side bicomponent nanofiber membrane with enhanced mechanical strength and good thermal stability. Nanomaterials.

[19] Zhang J., Li S., Ju D.D., Li X., Zhang J.C., Yan X., Long Y.Z., Song F. (2018). Flexible inorganic core-shell nanofibers endowed with tunable multicolor up conversion fluorescence for simultaneous monitoring dual drug delivery. Chem. Eng. J..

[20] Chen S., Liu G.S., He H.W., Zhou C.F., Yan X., Zhang J.C. (2019). Physical structure induced hydrophobicity analysed from electrospinning and coating Poly(vinyl butyral) (PVB) films. Adv. Condens. Matter Phys..

[21] Vohrer U., Tricka I., Bernhardt J., Oehr C., Brunner H. (2001). Plasma treatment: An increasing technology for paper restoration?. Surf. Coat. Technol..

[22] Yuan X., Jayaraman K., Bhattacharyya D. (2004). Effects of plasma treatment in enhancing the performance of woodfibre-polypropylene composites. Compos. Part A.

[23] Totolin M.I., Neamtu I. (2011). Positive findings for plasma polymer (meth) acrylate thin films in heritage protective applications. J. Cult. Herit..

[24] Yang J., Pu Y., Miao D., Ning X. (2018). Fabrication of durably superhydrophobic cotton fabrics by atmospheric pressure plasma treatment with a siloxane precursor. Polymers.

[25] Ioanid E.G., Rusu D., Dunca S. (2010). High-frequency plasma in heritage photo decontamination. Ann. Microbiol..

[26] Li Q., Xi S., Zhang X. (2014). Deacidification of paper relics by plasma technology. J. Cult. Herit..

[27] Dong Y., Li C., Wang Y., Zhou T. (2007). Anti-ultraviolet finish of cotton fabric by air low-temperature plasma. Prog. Text. Sci. Tech..

[28] Cheng C., Fang P., Zhu X., Geng S., Zhan R. (2005). Growth of SiOx films by dielectric barrier discharge (DBD) plasma enhanced chemical vapor deposition at atmospheric pressure. Chin. J. Vac. Sci. Tech..

[29] Palaskar S., Kale K.H., Nadiger G.S., Desai A.N. (2011). Dielectric barrier discharge plasma induced surface modification of polyester/cotton blended fabrics to impart water repellency using HMDSO. J. Appl. Polym. Sci..

[30] Li J., Yan L., Zhao Y., Zha F., Wang Q., Lei Z. (2015). One-step fabrication of robust fabrics with both-faced superhydrophobicity for the separation and capture of oil from water. Phys. Chem. Chem. Phys..

