# Supplementary material for: In Situ Surface Modification of Paper-Based Relics with Atmospheric Pressure Plasma Treatment for Preservation Purposes
Source: Polymers (Basel). 2019 May 2;11(5):786. doi: 10.3390/polym11050786 (PMC6572557; doi:10.3390/polym11050786)
Supplement: Supplementary file 1 [file polymers-11-00786-s001.zip › polymers-481613-SP/Figure S1.docx]

Supplementary materials

In situ surface modification of paper-based relics with atmospheric pressure plasma treatment for preservation purposes

Xu Yan ^1, 2, 3*^, Guo-Sai Liu ^1^, Jing Yang ^1,3^, Yi Pu^1^, Shuo Chen^1^, Hong-Wei He^1^, Conger Wang^4^, Yun-Ze Long^1, 2, 5^ and Shouxiang Jiang ^3,^*


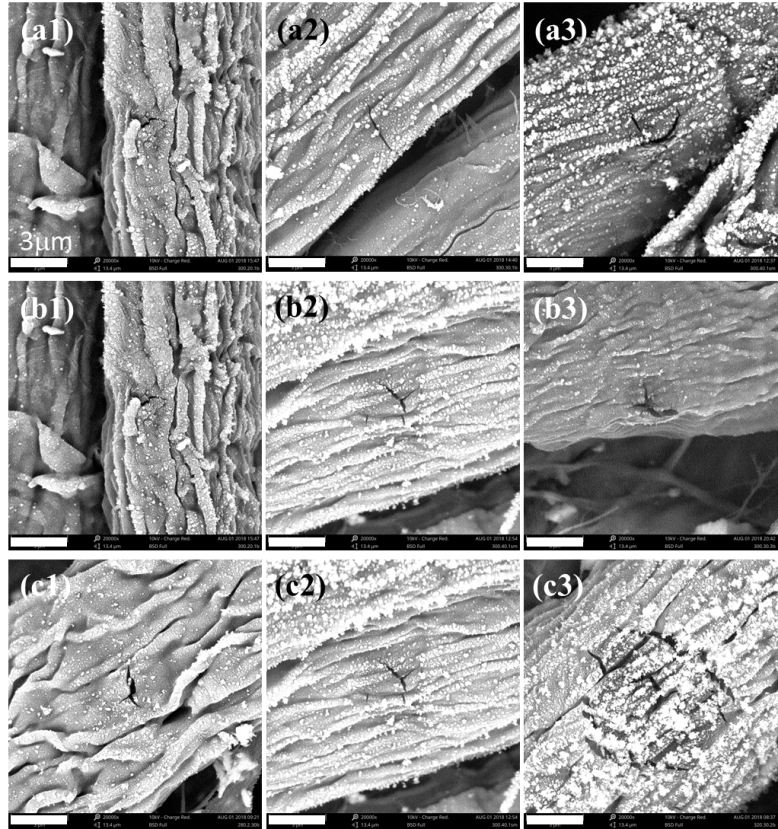


Figure S1. The SEM images of the plasma treated paper relic with different precursor value (a1) 20 g/h, (a2) 30 g/h and (a3) 40 g/h under 300 V and one repeat treatment times; different repeat treatment numbers 1-3 (b1)-(b3), respectively under 300 V and precursor value 30 g/h; different voltage (c1) 280 V, (c2) 300 V, (c3) 320 V with precursor value 30 g/h and two repeat treatment times
